# The prevalence of cardiac autonomic neuropathy in prediabetes: a systematic review

**DOI:** 10.1007/s00125-020-05316-z

**Published:** 2020-11-09

**Authors:** Aikaterini Eleftheriadou, Scott Williams, Sarah Nevitt, Emily Brown, Rebecca Roylance, John P. H. Wilding, Daniel J. Cuthbertson, Uazman Alam

**Affiliations:** 1 Department of Cardiovascular & Metabolic Medicine, Institute of Life Course and Medical Sciences, University of Liverpool, Liverpool, UK; 2 Department of Biostatistics, University of Liverpool, Liverpool, UK; 3 Edge Hill University Library, Aintree University Hospital NHS Foundation Trust, Liverpool, UK; 4 Obesity and Endocrinology Research, Department of Cardiovascular & Metabolic Medicine, Institute of Life Course and Medical Sciences, University of Liverpool, Liverpool, UK; 5 Division of Diabetes, Endocrinology and Gastroenterology, Institute of Human Development, University of Manchester, Manchester, UK; 6 Pain Research Institute and Department of Cardiovascular & Metabolic Medicine, Institute of Life Course and Medical Sciences, University of Liverpool and Aintree University Hospital NHS Foundation Trust, Liverpool, UK; 7 Department of Diabetes and Endocrinology, Liverpool University Hospital NHS Trust, Liverpool, UK

**Keywords:** Cardiac autonomic neuropathy, Lifestyle intervention, Obesity, Prediabetes, Systematic review, The metabolic syndrome

## Abstract

**Aims/hypothesis:**

Cardiac autonomic neuropathy (CAN) is independently associated with silent myocardial ischaemia, major cardiovascular events, myocardial dysfunction and cardiovascular mortality. Several studies have highlighted the increased prevalence of CAN in prediabetes (impaired glucose tolerance and/or impaired fasting glucose). Considering the exponential rise of prediabetes, we aimed to determine the prevalence of CAN through a systematic literature review.

**Methods:**

This systematic review was registered with PROSPERO (CRD42019125447). An electronic literature search was performed using MEDLINE, EMBASE, PubMed, Web of Science, Scopus and Cochrane databases. Published full text, English language articles that provide CAN prevalence data of studies in individuals with prediabetes and aged over 18 years were included. Prevalence data for normal glucose tolerance and diabetes were also extracted from the selected articles, if present. All articles were screened by two independent reviewers using a priori criteria. Methodological quality and risk of bias were evaluated using a critical appraisal tool.

**Results:**

Database searches found 4500 articles; subsequently, 199 full text articles were screened, 11 of which fulfilled the inclusion criteria (4431 total participants, 1730 people with prediabetes, 1999 people with normal glucose tolerance [NGT] and 702 people with predominantly type 2 diabetes). Six of the selected studies reported definite CAN prevalence data (9–39%). Only a single large population-based study by Ziegler et al (KORA S4 study, 1332 participants) determined definite CAN based on two or more positive autonomic function tests (AFTs), with a mean prevalence of 9% in all prediabetes groups (isolated impaired glucose tolerance 5.9%; isolated impaired fasting glucose 8.1%; impaired fasting glucose plus impaired glucose tolerance 11.4%), which was higher than NGT (4.5%). This study is most likely to provide a reliable population-specific estimate of CAN in prediabetes. There was a higher than expected prevalence of CAN in prediabetes (9–38%) when compared with normal glucose tolerance (0–18%) within the same studies (*n* = 8). There was a wide prevalence of possible CAN based on one positive AFT (*n* = 5). There was heterogeneity between the studies with variations in the definition of CAN, methodology and characteristics of the populations, which likely contributed to the diversity of prevalence estimates. The overall risk of bias was low.

**Conclusions/interpretation:**

There is a higher than expected prevalence of CAN in prediabetes. Early detection of CAN in prediabetes through population screening needs careful consideration in view of the excess morbidity and mortality risk associated with this condition.

**Figure:**
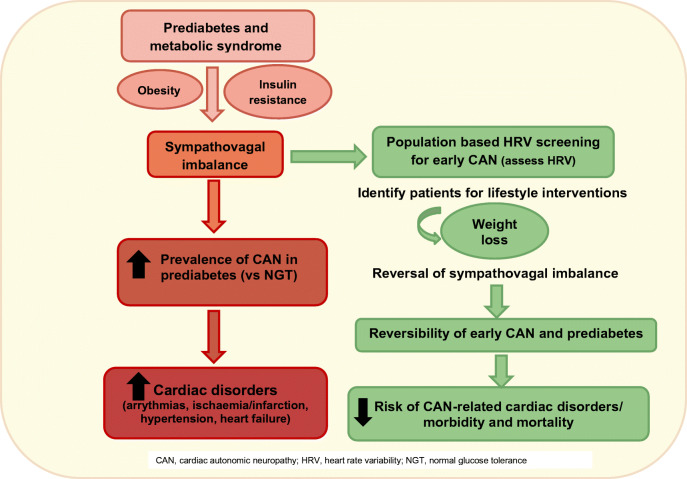
Graphical abstract

**Supplementary Information:**

The online version of this article (10.1007/s00125-020-05316-z) contains peer-reviewed but unedited supplementary material, which is available to authorised users.

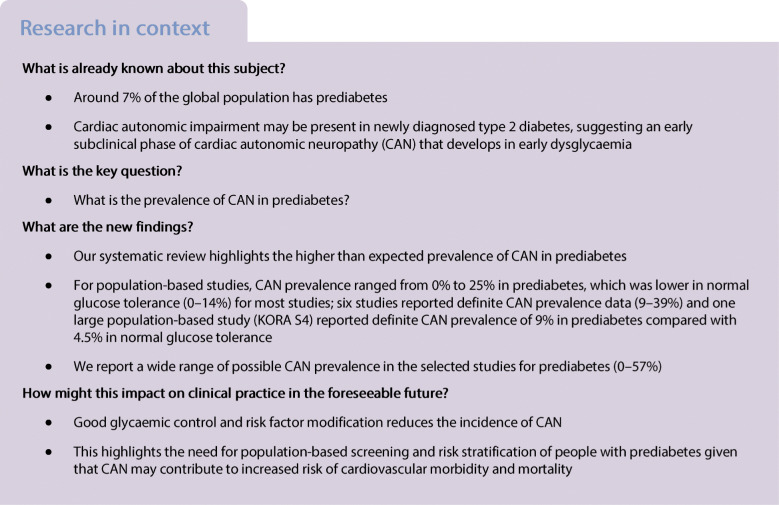



## Introduction

The projected prevalence of prediabetes (people with impaired glucose tolerance [IGT] and/or impaired fasting glucose [IFG]) is similar to type 2 diabetes and both are becoming major global epidemics [1, 2]. An estimated 34% of adults in the USA (equivalent to ~84.1 million people) had prediabetes in 2015 [3] whereas the IDF projects an increase in prevalence of prediabetes to 471 million globally by 2035 [2]. The prevalence rates of prediabetes have markedly increased in England from ~12% to ~35% from 2003 to 2011 [4].

Prediabetes is associated with classical microvascular and macrovascular complications suggesting a deleterious environment for microvasculature [5]. The Whitehall study showed that IGT conferred an increased risk of large vessel disease with a doubling of CHD mortality rate [6]. Microalbuminuria is increased in people with IGT compared with healthy individuals [7]. Indeed, there is also a high prevalence of peripheral neuropathy in prediabetes [8]. Asghar et al showed the prevalence of small-fibre neuropathy was ~40% in prediabetes thus suggesting early nervous system pathology [9]. Abnormalities of autonomic function with impaired sympathovagal balance may coexist with, or even predict subsequent development of, microvascular complications including diabetic neuropathy [10].

Cardiac autonomic neuropathy (CAN) is a serious but under-recognised complication of diabetes, resulting in cardiac denervation and thus increased morbidity and mortality risk [11–14]. The prevalence of CAN in populations with diabetes has been reported to be as high as 90% [15]. Longitudinal studies in CAN have shown 5 year mortality rates in type 1 and type 2 diabetes of 16–50%, with a high proportion attributed to cardiac sudden death [16]. The Rochester Diabetic Neuropathy Study showed that in all cases of sudden death, with or without diabetes, there was severe CAN or left ventricular dysfunction [17, 18]. This finding is supported by studies showing that CAN is independently associated with a higher mortality rate, when adjusting for cardiovascular covariates (1, 27, 28). Moreover, higher mortality rates are observed in individuals with both recent myocardial infarction and abnormal heart rate variability (HRV) [19, 20]. CAN is associated with major cardiovascular events such as ventricular tachycardia/fibrillation, need for coronary revascularisation and excess cerebrovascular disease [21].

Importantly, cardiac autonomic impairment occurs in the early stages of diabetic metabolic dysfunction with progressive worsening of cardiac autonomic function over time [22, 23]. Risk factors for CAN include prediabetic and diabetic range dysglycaemia, dyslipidaemia, hypertension, elevated BMI and increased waist circumference [24–26]. A number of studies have considered the association between autonomic dysfunction in prediabetes and the metabolic syndrome and have reported an increased prevalence of CAN compared with healthy people [25–29], although this has been contradicted in other published data [30].

There have been no systematic reviews undertaken to assess the prevalence of CAN in prediabetes. Our aim was to systematically review the epidemiology of CAN and determine its prevalence in prediabetes in published literature through a systematic literature review.

## Methods

### Search strategy

Following the international standard PRISMA guidelines, the protocol for this review specifying the objectives, inclusion criteria and methods of analysis is registered with PROSPERO (registration ID CRD42019125447). Electronic searches were performed to identify articles reporting the prevalence of CAN in prediabetes, using the following databases: MEDLINE (access via OVID); EMBASE (access via OVID); PubMed; Web of Science; Scopus; and Cochrane databases. As the a priori protocol also included extraction of studies of the metabolic syndrome, this was also included in the initial strategy. A qualified medical librarian (RR) and a second trained researcher (AE) independently conducted database searches. Searches were restricted to English language from inception to June 2019. Combinations of pre-specified search terms were used (Table 1). Results from the databases were merged using EndNote to facilitate the removal of duplicates. Reference lists of studies, review articles and systematic reviews were manually reviewed to identify any additional studies.

**Table 1:** Search terms

Prediabetes or the metabolic syndrome related terms	CAN related terms
‘Pre-diabetes or prediabetes’	‘Cardiac autonomic neuropathy’
‘Impaired glucose tolerance’	‘Cardiovascular autonomic dysfunction’
‘IGT’	‘Autonomic neuropathy’
‘Impaired fasting glucose’	‘Abnormal heart rate variability’
‘IFG’	‘Autonomic Function Tests’
‘Metabolic syndrome’	‘Cardiac autonomic function tests’,
‘MS’	‘Cardiovascular autonomic reflex tests’,
‘Insulin resistance syndrome’	‘Borderline autonomic function tests’
‘Metabolic syndrome X’	‘Definite CAN’
‘Dysmetabolic syndrome’	‘Mild cardiac autonomic neuropathy’,
‘Syndrome X’	‘Moderate cardiac autonomic neuropathy’,
	‘Severe cardiac autonomic neuropathy’
	‘30:15 ratio’
	‘E/I’
	‘E:I’
	‘Orthostatic hypotension’
	‘Postural hypotension’
	‘Neuropathy’
	‘Diabetic neuropathy’
	‘Valsalva ratio’
	‘Heart rate response to deep breathing’
	‘Heart rate response to Valsalva manoeuvre’
	‘Resting heart rate variability’
	‘Heart rate response to standing’
	‘Prolonged QT interval’
	‘Sympathetic nervous system’
	‘SNS overactivity’
	‘Parasympathetic nervous system denervation’
	‘PNS denervation’
	‘Vagus nerve denervation’
	‘Resting tachycardia’
	‘Palpitations’
	‘Syncope’
	‘Sudomotor function’

### Inclusion/exclusion criteria

A priori inclusion/exclusion criteria were used to select the final article.

Studies were included if they met the following criteria:

1. case controls, cohorts and observational studies displaying prevalence data for CAN in prediabetes; CAN was defined according to the Toronto Diabetic Neuropathy Consensus Panel as two or more positive tests indicating definite CAN, while possible CAN was defined using a single positive test [14];
2. included adults ≥18 years old who had prediabetes defined either by the WHO [31, 32] or ADA criteria [33];
3. were full-text publications.

Studies were excluded if they met the following criteria:

1. not an original research manuscript;
2. not a human study;
3. not conducted in adults (≥18 years);
4. participants did not have prediabetes;
5. did not report prevalence figures of CAN within prediabetes;
6. were not written in English.

**Fig. 1:**
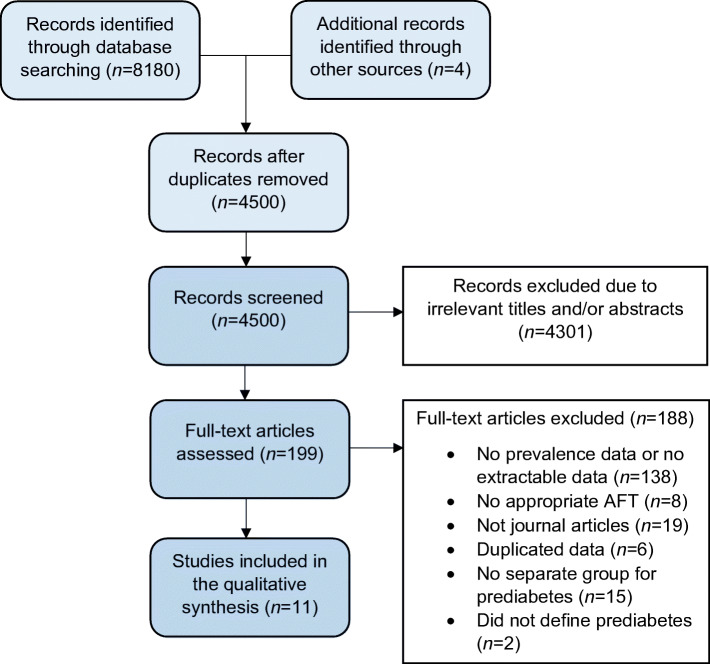
PRISMA flowchart demonstrating the article screening process

Two authors (AE and SW) independently screened the titles and abstracts from the literature search from all databases mentioned. All eligible articles were selected for full critique. If there was any doubt regarding the eligibility of any given study, the paper was included for critique of the full text. Two authors (AE and SW) independently assessed the full text articles, using the inclusion/exclusion criteria. The senior author (UA) decided on exclusion or inclusion, in the event of disagreement. The process of screening and selection for inclusion were recorded using a PRISMA flowchart (Fig. 1).

### Data extraction and quality assessment

Before data extraction and quality assessment, UA screened all articles in order to confirm their eligibility within this study. The data from the final selected articles were extracted independently using a standardised spreadsheet by two authors (AE and SW). Study characteristics, methodology data and results from studies were extracted. Extraction of the studies’ first author, study name, year of publication, country, type of study and setting was completed. Subsequently, detailed study characteristics, clinical and demographics data were extracted (e.g. sample size and population data, age/sex, definition of prediabetes, definition and diagnostics of CAN, etc). The combined extracted data was reviewed by UA to ensure accuracy of the data extraction.

### Controls and comparators

Controls and comparators included participants with normal glucose tolerance (NGT) or diabetes. These data were extracted if these groups were included in the selected studies; however, lack of either a control group or diabetes group was not an exclusion criterion.

### Critical appraisal

A critical appraisal tool was used on the final studies selected to be reviewed, specifically addressing the external and internal validity of the articles. AE and SW independently evaluated the quality of the studies using the validated tool developed by Munn et al [34]. Nine questions were posed for each article; a score of 0 or 1 was recorded representing a ‘yes’ or ‘no’ response, respectively, determining confounding, selection bias, bias related to measurement and data analysis. A total score between 0 and 3 was considered low risk, 4–6 was moderate risk and ≥7 was high risk of bias, as defined by the authors of the critical appraisal tool. Any discrepancies in the risk of bias were put forward to the senior author (UA) for a final decision.

### Definition of CAN and analysis of subgroups

Definite CAN is defined as two or more positive cardiac autonomic tests as per the definitions of the Toronto Diabetic Neuropathy Expert Group [14]. An a priori decision was made to undertake subgroup analysis of definite CAN vs possible CAN. Methodological variables that may have affected the prevalence in any specific study were extracted (e.g. ethnicity, sex, BMI, age and assessment of autonomic neuropathy).

### Data analysis

All final selected articles were included in the systematic review. Clinical heterogeneity of the studies was assessed by comparing study designs and participant characteristics. Statistical heterogeneity was assessed using the *I*^2^ statistic [35]. If clinical or statistical heterogeneity was deemed to be too high (e.g. *I*^2^ >90%) to provide a reliable or useful pooled prevalence estimate, meta-analysis may have been conducted using the generic inverse variance method and conducted with random-effects due to anticipated clinical heterogeneity. However, the heterogeneity was considered clinically high (but <90%), therefore a formal meta-analysis was not conducted. Individual study results are presented in tabular format without the summary pooled prevalence estimate and included in the electronic supplementary material (ESM), and results are described narratively. Prevalence data expressed as a proportion of people with CAN were extracted or calculated from the data available in the studies. A funnel plot was created to show possible bias within the prevalence results (ESM Fig. 1).

All analyses and figure production, including the forest plots, were undertaken using Review Manager 5.4 (Cochrane Collaboration, York, UK). Data in the tables are expressed as mean (±SD or range when applicable).

## Results

### Search results

After the removal of duplicates a total of 4500 articles were generated from the electronic database and manual reference searches. A PRISMA flowchart was completed displaying the article exclusions at each stage of screening (Fig. 1). The titles and abstracts (*n* = 4500) were screened using the inclusion/exclusion criteria, excluding 4301 articles. Analysis of 199 full texts was performed, 11 of which fulfilled the inclusion criteria, and data were extracted (Tables 2, 3 and 4).

**Table 2:** Participant demographic characteristics information from all final selected articles

Study	Country	Primary care or hospital based	Sample size	Study group	Group size	Age, years	Sex, *n* female/*n* male	Male, %
Ziegler et al, 2015 [25]	Germany	Primary care/ population based	1332	iIFG	336	63 (58–68)	128/208	62
				iIGT	72	65 (62–69)	44/28	39
				IFG+IGT	151	65 (61–70)	61/90	60
				NGT	565	63 (58–68)	328/237	42
				kDM	130	65 (61–69)	60/70	54
				nDM	78	66 (61–71)	33/45	58
Dimova et al, 2020 [41]	Bulgaria	Primary care/population based	87	PreDM	35	44.8 ± 10.2	19/16	46
				NGT	35	45.5 ± 14.1	19/16	46
				nDM	17	48.0 ± 8.5	10/7	41
Laitinen et al, 2011 [38]	Finland	Primary care/population based	268	IGT	268	62 ± 7	177/91	34
Wu et al, 2009 [42]	China	Primary care/population based	1638	PreDM	412	49.5 ± 14.2	218/194	47
				NGT	1069	39.4 ± 14.0	568/501	47
				DM	157	57.7 ± 12.8	67/90	57
Zimmerman et al, 2018 [40]^a^	Sweden	Primary care/population based	119	IGT	29	NA	9/20	69
				NGT	39	NA	18/21	54
				(T2)DM	51	NA	15/36	71
Dimova et al, 2017 [36]	Bulgaria	Primary care and hospital based	478	PreDM	227	NA	NA	NA
				IFG	125	51.7 ± 12.1	71/54	43
				IGT	102	49.2 ± 13.5	71/31	30
				NGT	130	46.6 ± 11.4	82/48	37
				(T2)DM	121	54.4 ± 11.5	54/67	55
Balbinot et al, 2012 [43]	Brazil	Hospital based	79	PreDM	13	56.8 ± 12.6	10/3	23
				NGT	37	45.1 ± 14	21/16	43
				(T2)DM	29	59.9 ± 9.4	10/9	31
Dinh et al, 2011 [37]	Germany	Hospital based	145	IGT	48	NA	NA	NA
				NGT	45^b^	57 ± 11	NA	NA
				(T2)DM	51	NA	NA	NA
Callaghan et al, 2020 [44]	USA	Hospital based	184	Obese PreDM	56	44.7 ± 11.4	46/10	18
				Lean NGT	46	44.1 ± 12.1	38/8	17
				Obese NGT	33	40.2 ± 10.7	25/8	24
				Obese DM	49	48.9 ± 10.4	34/15	31
Putz et al, 2013 [39]	Hungary	Hospital based/primary care	115	IGT	75	58.7 ± 11.1	41/34	45
				NGT	40	55.1 ± 10.0	23/17	43
Kamel et al, 2014 [45]	Australia	NA	52	IGT	8	NA	NA	NA
				NGT	26^b^	49	13/13	50
				DM	18	NA	NA	NA

Age data are shown as mean±SD or median (range)

^a^Data from the starting point of the cohort study was used; however, the authors presented the starting point data only for the participants that remained in the cohort study until the latest follow-up

^b^Not included in the total population for this review as no prevalence data available for this population

Prediabetes groups: IFG, IGT, iIFG and iIGT

DM, diabetes mellitus; FPG, fasting plasma glucose; kDM, known diabetes mellitus; NA, information is not available; nDM, new diabetes mellitus; PreDM, prediabetes; (T2)DM, type 2 diabetes mellitus

**Table 3:** Participant metabolic characteristics from all final selected articles

Study	Subgroup	FPG, mmol/l (mg/dl)	OGTT, mmol/l	HbA_1c_, % (mmol/mol)	Systolic BP, mmHg	Diastolic BP, mmHg	Triacylglycerols, mmol/l (mg/dl)	Cholesterol, mmol/l	BMI, kg/m^2^
Ziegler et al, 2015 [25]	iIFG	NA	NA	5.6 (5.4–5.8) (37.7 [35.5–39.9])	NA	NA	1.33 (0.98–1.81)	NA	28.2 (25.8–30.8)
	iIGT	NA	NA	5.5 (5.3–5.8) (36.6 [34.4–39.9])	NA	NA	1.30 (1.03–1.85)	NA	29.4 (26.1–32.0)
	IFG+IGT	NA	NA	5.7 (5.5–6.0) (38.8 [36.6–42.1])	NA	NA	1.45 (1.16–2.11)	NA	29.1 (27.4–31.9)
	NGT	NA	NA	5.5 (5.3–5.8) (36.6 [34.4–39.9])	NA	NA	1.16 (0.84–1.62)	NA	26.9 (24.7–29.5)
	kDM	NA	NA	6.8 (6.2–7.9) (50.8 [44.3–62.8])	NA	NA	2.23 (1.53–3.15)	NA	31.2 (28.1–34.3)
	nDM	NA	NA	6.1 (5.7–6.4) (53.2 [38.8–46.4])	NA	NA	1.65 (1.30–2.25)	NA	29.9 (27.9–33.0)
Dimova et al, 2020 [41]	PreDM	6.4 ± 0.5	7.6 ± 1.9	5.7 ± 0.7 (39 ± 6)	125 ± 12	79 ± 9	1.7 (0.9–2.4)	5.3 ± 1.0	33.3 ± 5.9
	NGT	5.5 ± 0.4	5.2 ± 1.2	5.6 ± 0.3 (38 ± 3)	122 ± 14	78 ± 11	1.5 (1.0–2.2)	5.6 ± 1.3	28.7 ± 6.5
	nDM	9.3 ± 2.6	14.6 ± 4.9	7.7 ± 1.4 (61 ± 15)	127 ± 19	81 ± 13	1.9 (1.6–2.7)	5.6 ± 0.9	33.2 ± 6.8
Laitinen et al, 2011 [38]	IGT	6.1 ± 0.7	8.2 ± 2.3	5.5 ± 0.4 (36.6 ± 4.4)	133 ± 18	79 ± 10	1.5 ± 0.6	5.4 ± 0.9	30.3 ± 5.4
Wu et al, 2009 [42]	PreDM	5.6 ± 0.5	NA	5.1 ± 0.6 (32.2 ± 6.6)	124.5 ± 21.0	74.7 ± 10.9	1.5 ± 0.9	5.2 ± 1.0	24.7 ± 3.8
	NGT	4.9 ± 0.4	NA	4.9 ± 0.5 (30.1 ± 5.5)	114.4 ± 17.5	69.7 ± 9.6	1.3 ± 1.0	4.9 ± 1.1	23.0 ± 3.3
	DM	8.7 ± 3.4	NA	7.5 ± 2.3 (58.5 ± 25.3)	135.8 ± 24.9	78.7 ± 11.1	2.3 ± 3.6	5.3 ± 1.4	25.8 ± 3.5
Zimmerman et al, 2018 [40]^a^	IGT	NA	NA	5.5 (5.3–5.6) (36.6 [34.4–37.7])	NA	NA	1.35 (0.85–1.65)	5.1 (4.3–5.9)	27.1 ± 5.4
	NGT:	NA	NA	5.4 (5.2–5.5) (35.5 [33.3–36.6])	NA	NA	1.09 (0.82–1.52)	5.9 (5.2–6.4)	25.8 ± 3.6
	(T2)DM	NA	NA	7.2 (6.5–7.9) (55.2 [47.5–62.8])	NA	NA	1.47 (1.08–1.99)	4.7 (4.3–5.1)	29.7 ± 4.3
Dimova et al, 2017 [36]	IFG	6.5 ± 0.3	6.1 ± 1.1	5.9 ± 0.4 (41.0 ± 4.4)	126 ± 14	77 ± 11	1.6 ± 1.4	5.2 ± 1.2	31.3 ± 5.8
	IGT	6.2 ± 0.8	9.1 ± 1.1	5.9 ± 0.4 (41.0 ± 4.4)	125 ± 14	77 ± 11	1.9 ± 1.2	5.4 ± 1.2	31.6 ± 6.1
	NGT	5.4 ± 0.4	5.3 ± 1.3	5.6 ± 0.3 (37.7 ± 3.3)	121 ± 14	76 ± 10	1.3 ± 0.8	5.2 ± 1.2	29.1 ± 6.2
	(T2)DM	10.1 ± 4.4	15.2 ± 6	8.2 ± 2.4 (66.1 ± 26.4)	129 ± 18	80 ± 11	2.2 ± 1.8	5.5 ± 1.2	32.5 ± 6.2
Balbinot et al, 2012 [43]	PreDM	NA	NA	NA	130.5 ± 6.2	83.5 ± 6.3	NA	NA	30.0 ± 2.8
	NGT	NA	NA	NA	121.7 ± 9.0	79.7 ± 8.8	NA	NA	25.3 ± 3.8
	(T2)DM	NA	NA	NA	131.7 ± 8.0	85.9 ± 7.9	NA	NA	27.1 ± 4.0
Dinh et al, 2011 [37]		NA	NA	NA	NA	NA	NA	NA	NA
Callaghan et al, 2020 [44]	Obese PreDM	5.4 ± 0.6 (98.5 ± 10.3)	7.0 ± 1.7 (126.6 ± 31.4)	5.7 ± 0.3 (39.1 ± 3.6)	131.5 ± 15.6	74.2 ± 13.3	1.4 ± 0.6 (122.7 ± 48.2)	NA	46.8 ± 7.1
	Lean NGT:	4.7 ± 0.4 (84.9 ± 6.3)	4.9 ± 1.1 (89.0 ± 19.5)	NA	108.7 ± 10.2	66.2 ± 9.5	0.8 ± 0.3 (71.2 ± 22.7)	NA	23.0 ± 2.0
	Obese NGT	4.9 ± 0.3 (88.2 ± 5.7)	5.3 ± 1.0 (96.3 ± 17.8)	5.3 ± 0.3 (34.4 ± 2.9)	126.2 ± 16.0	72.9 ± 12.5	1.2 ± 0.5 (104.5 ± 42.8)	NA	48.5 ± 8.3
	Obese DM	7.2 ± 2.4 (129.5 ± 43.4)	8.7 ± 3.6 (156.5 ± 64.3)	7.4 ± 1.5 (57.7 ± 16.5)	131.2 ± 12.8	72.9 ± 88	1.9 ± 1.5 (160 ± 129.9)	NA	45.0 ± 6.8
Putz et al, 2013 [39]	IGT	5.6 ± 0.6	8.7 ± 1.0	6.0 ± 0.3 (42.1 ± 2.0)	126 ± 12	75 ± 7	1.3 (1.0–1.7)	5.0 ± 1.1	29.9 ± 4.7
	NGT	4.9 ± 0.5	4.9 ± 0.5	5.0 ± 0.5 (31.1 ± 3.1)	117 ± 10	71 ± 6	1.3 (0.9–1.8)	5.1 ± 1.0	25.1 ± 3.9
Kamel et al, 2014 [45]	IGT	NA	NA	NA	NA	NA	NA	NA	NA
	NGT	NA	NA	NA	NA	NA	NA	NA	NA
	DM	NA	NA	NA	NA	NA	NA	NA	NA

Data are shown as mean±SD or median (range)

^a^Data from the starting point of the cohort study was used; however, the authors presented the starting point data only for the participants that remained in the cohort study until the latest follow-up

Prediabetes groups: IFG, IGT, iIFG and iIGT

DM, diabetes mellitus; FPG, fasting plasma glucose; kDM, known diabetes mellitus; NA, information is not available; nDM, new diabetes mellitus; PreDM, prediabetes; (T2)DM, type 2 diabetes mellitus

**Table 4:** CAN prevalence in prediabetes from all final selected articles

Author	Country	Sample size	Study group	Group size	Age, years	Sex, *n* female/ *n* male	No. of AFTs used for diagnosis	AFT tests	No. of participants with CAN	Prevalence, %
Ziegler et al, 2015 [25]	Germany	1332	iIFG	336	63 (58–68)	128/208	≥2	Linear HRV analysis (time and frequency domain) and non-linear HRV analysis derived from non-linear dynamics	27	8.1
			iIGT	72	65 (62–69)	44/28			4	5.9
			IFG+IGT	151	65 (61–70)	61/90			17	11.4
			NGT	565	63 (58–68)	328/237			25	4.5
			kDM	130	65 (61–69)	60/70			23	17.5
			nDM	78	66 (61–71)	33/45			9	11.7
			DM	NA	NA	NA			32	15.4
Dimova et al, 2020 [41]	Bulgaria	87	PreDM	35	44.8 ± 10.2	19/16	≥2	АNX-3.0 method that computes sympathetic and parasympathetic activity non-invasively, separately and simultaneously based on cardiorespiratory synchronisation at rest and Ewing tests^a^: deep breathing challenge, Valsalva challenge and stand-up challenge	3	8.6
			NGT	35	45.5 ± 14.1	19/16			2	5.7
			nDM	17	48.0 ± 8.5	10/7			4	23.5
Laitinen et al, 2011 [38]	Finland	268	IGT	268	62 ± 7	177/91	1	Deep breathing test; active orthostatic test; expiration/inspiration (E/I) ratio^a^	67	25
Wu et al, 2009 [42]	China	1638	PreDM	412	49.5 ± 14.2	218/194	1	Orthostatic hypotension, BP and heart rate variability after standing^a^	73	17.7
			NGT	1069	39.4 ± 14.0	568/501			148	13.8
			DM	157	57.7 ± 12.8	67/90			40	25.5
Zimmerman et al, 2018 [40]	Sweden	119	IGT	29	NA	18/15	1	Expiration/inspiration (E/I) ratio	0	0
			NGT	39	NA	9/11			0	0
			(T2)DM	51	NA	15/19			0	0
Dimova et al, 2017 [36]	Bulgaria	478	PreDM	227	NA	NA	≥2	HRV at rest and during deep breathing, Valsalva challenge, standing challenge^a^	NA	19.8
			IFG	125	51.7 ± 12.1	71/54			17	13.2
			IGT	102	49.2 ± 13.5	71/31			21	20.6
			NGT	130	46.6 ± 11.4	82/48			16	12.3
			(T2)DM	121	54.4 ± 11.5	54/67			39	32.2
Balbinot et al, 2012 [43]	Brazil	79	PreDM	13	56.8 ± 12.6	10/3	≥2	HRV tests comprised three spectral indices (in the frequency domain) and four Ewing tests; including the Valsalva manoeuvre, orthostatic test, deep breathing test^a^ and orthostatic hypotension test	5	38.5
			NGT	37	45.1 ± 14	21/16			3	8.1
			(T2)DM	29	59.9 ± 9.4	10/9			16	55.2
Dinh et al, 2011 [37]	Germany	145	IGT	48	NA	NA	≥2	HRV at rest; spectral power in the very low-frequency band; spectral power in the low-frequency band; HRV during deep breathing; maximum/minimum 30:15 ratio; Valsalva ratio; postural change in systolic blood pressure^a^	6	12.5
			NGT	45^c^	57 ± 11				NA	NA
			(T2)DM	51	NA				15	29
Callaghan et al, 2020 [44]	USA	184	Obese PreDM	56	44.7 ± 11.4	46/10	1	Expiration/inspiration (E/I) ratio	12	21.4
			Lean NGT	46	44.1 ± 12.1	38/8			2	4.4
			Obese NGT	33	40.2 ± 10.7	25/8			6	18.2
			Obese DM	49	48.9 ± 10.4	34/15			16	31.9
Putz et al, 2013 [39]	Hungary	115	IGT	75	58.7 ± 11.1	41/34	1 or ≥2	HRV during deep	43	57.3
			NGT	40	55.1 ± 10.0	23/17		Breathing, standing (30/15 ratio) and Valsalva manoeuvre (Valsalva ratio), BP response to standing and sustained handgrip^c^	0	0
Kamel et al, 2014 [45]	Australia	52	IGT	8	NA	NA	≥2	HRV response to Valsalva manoeuvre. Heart rate variation during deep breathing. BP response to standing, BP response to sustained handgrip was performed by first determining the maximum voluntary contraction using a handgrip dynamometer^c^	2	25
			NGT	26^b^	49	13/13			NA	NA
			DM	18	NA	NA			5	27.8

Age data are shown as mean±SD or median (range)

^a^Used part or all of the reference standard Ewing and O’Brien protocol. The Ewing and O’Brien battery of tests were a number of decades ago to assess cardiovascular autonomic function and are non-invasive, simple, safe, reliable, reproducible and standardised. They primarily evaluate parasympathetic dysfunction, which is the primary aspect of the cardiac autonomic system that exhibits dysfunction in CAN/diabetes. For further reading on CAN diagnosis and testing, please refer to: https://www.ncbi.nlm.nih.gov/pmc/articles/PMC3822331/#

^b^Not included in the total population for this review as no prevalence data available for this population

^c^the data from the starting point of the cohort study was used; however, the authors presented the starting point data only for the participants that remained in the cohort study until the latest follow-up

Prediabetes groups: IFG, IGT, iIFG and iIGT

DM, diabetes mellitus; kDM, known diabetes mellitus; NA, information is not available; nDM, new diabetes mellitus; PreDM, prediabetes; (T2)DM, type 2 diabetes mellitus

### Study characteristics

#### Summary of studies

The majority of studies were carried out in European populations (*n* = 7) [25, 36–41]; other locations were China (*n* = 1) [42], Brazil (*n* = 1) [43], USA (*n* = 1) [44] and Australia (*n* = 1) [45]. The studies sample sizes ranged from 52 to 1638 participants [25, 36–47]. All studies included tests of cardiac autonomic function. Only two small studies (total participants *n* = 150) of CAN prevalence in the metabolic syndrome met the inclusion criteria. As their inclusion may have provided an unrepresentative estimate due to the paucity of data, these results have been included in the ESM (ESM Table 1 and 2) only and will not be considered any further in the main manuscript.

#### Study design and participants

Eight studies were cross sectional [25, 36, 37, 39, 41–44], two were cohort studies [38, 40] and one was a prospective observational study [45]. CAN prevalence data were presented for a total of 1730 participants with prediabetes, 1999 participants with NGT and 702 participants with predominantly type 2 diabetes. The mean age of participants in the studies varied from 39 years to 65 years [25, 36–45]. In general, there was similar recruitment of participants based on sex. Five studies defined CAN as one abnormal autonomic function test (AFT) [38–40, 42, 44], whereas six studies required two or more abnormal tests [25, 36, 37, 41, 43, 45]. Eight studies did not report the precise method of participant recruitment [36–39, 41, 43–45] and none of the studies included sample size calculation. CVD characteristics of the recruited participants varied between the selected studies. Five studies excluded participants suffering from ischaemic heart disease, [25, 41, 43–45] five studies included participants with ischaemic heart disease [36–39, 42] and one study did not specify [40].

CAN prevalence was reported for NGT groups in eight studies [25, 36, 39–44] and for diabetes groups in ten studies [25, 36, 37, 40–45].

### Prevalence of CAN in prediabetes

Data from the 11 final selected articles was used to investigate the prevalence of CAN in prediabetes. Six articles presented definite CAN prevalence data (≥2 abnormal AFTs; 890 participants, prevalence range 9–38.5%) [25, 36, 37, 41, 43, 45] while five articles presented possible CAN prevalence data (840 participants, prevalence range 0–57%) [38–40, 42, 44] in people with prediabetes. When reviewing studies based on the recruitment method (population/primary care-based vs hospital based), prevalence of definite CAN in population-based studies of prediabetes was only undertaken in a single study and was 9% [25]. The prevalence of definite and possible CAN in larger population-based studies ranged between 9% (*n* = 559) [25] and 17% (*n* = 412) [42] (ESM Fig. 2).

The majority of the studies reported a prevalence of between 20% and 40% (*n* = 3) [38, 39, 44] for possible CAN. Larger population-based studies showed higher prevalence of definite and possible CAN in prediabetes compared with NGT. The study of Ziegler et al [25] was the only large sized population-based study (*n* = 1332) detailing definite CAN prevalence, which was 9% in prediabetes and 4.5% in NGT. Dimova et al (2017) [36] (478 participants) utilised two AFTs for a positive test and reported a prevalence of 19.8% in prediabetes and 12.3% in NGT. Similarly, Wu et al [42] (*n* = 1638) used one AFT and showed a CAN prevalence of 18% in prediabetes and 14% in NGT. Table 4 and ESM Figs. 2a, 2b, 3 and 4 summarise data by displaying the prevalence figures for each study.

### Secondary analyses

In the NGT groups, overall CAN prevalence ranged from 0% to 18%. Definite CAN prevalence was reported in four studies (812 participants, prevalence range 4–18%) [25, 36, 41, 43]. The majority of the studies reported an overall prevalence <10% (*n* = 5) [25, 39–41, 43], although in the two adequately sized population-based studies [25, 42] the prevalence was 4.5% and 13.8% (ESM Fig. 3). Interestingly, Callaghan et al [44] found CAN prevalence in obese NGT to be 18.2%, which approached that in prediabetes (21.4%) suggesting that constituents of the metabolic syndrome play an important role in the pathogenesis.

Overall CAN prevalence (definite and possible) in the diabetes groups was more widely dispersed and ranged from 0% to 56% [25, 36, 37, 40–45] (diabetes prevalence: <10%, *n* = 1 [40]; 10–20%, *n* = 1 [25]; 20–30%, *n* = 4 [37, 41, 42, 45]; 30–40%, *n* = 1 [36]; >40%, *n* = 1 [43]) (ESM Fig. 4). Definite CAN prevalence in diabetes was reported in six studies (445 participants) [25, 36, 37, 41, 43, 45] and ranged from 15.4% to 55.2%. In population-based studies (*n* = 4) it ranged from 11.7% to 56.6% [25, 40–42].

### CAN definition and tests

The definition of CAN varied between the studies. CAN was defined as three abnormal AFTs (*n* = 1) [43], two abnormal AFTs (*n* = 5) [25, 36, 37, 41, 45] or one abnormal AFT (*n* = 5) [38–40, 42, 44]. A single study used gold standard HRV spectral power analysis to define CAN [25].

#### Different surrogate biomarkers for the criteria of CAN among the studies

All of endpoints used HRV response to physical manoeuvres (*n* = 11), according to Ewing’s protocol, including deep breathing HRV (*n* = 9) [36–41, 43–45], Valsalva challenge HRV (*n* = 6) [36, 37, 39, 41, 43, 45] and HRV response to standing challenge (*n* = 8) [36, 37, 39, 41–43, 45, 46]. Additionally, spectral HRV analyses were also utilised (*n* = 4) [25, 37, 41, 43]. Study CAN definitions are detailed in ESM Table 3.

### Risk of bias

Evaluation of bias and article quality showed all studies had a score suggestive of a low risk of bias. The median (IQR) risk of bias score was low at 1.6 (1.0) (out of 9) (ESM Table 4).

A funnel plot was created which showed no clear evidence of asymmetry, therefore it is unlikely that publication bias is present within this review (ESM Fig. 1).

## Discussion

The primary finding of this systematic review of 11 studies involving 1730 participants with prediabetes [36–39, 42, 43, 45] is the high prevalence of CAN in studies reporting prevalence based on the Toronto criteria. Prevalence of definite CAN was 9% in the sole population-based study of prediabetes, which determined CAN based on two or more AFTs [25]. The prevalence of definite and possible CAN in adequately sized population-based studies ranged between 9% and 17.7% and was greater than populations with NGT reported within the same studies [25, 42]. Unfortunately, a true estimate of population-level prevalence was not feasible due to the heterogeneous nature of the studies. There is a clear need to ascertain a true estimate of definite CAN using reference standard AFTs in a future large primary care-based study, primarily to ascertain levels of comorbidity in a prediabetes population with longitudinal evaluation. The results of this systematic review support the hypothesis that autonomic dysfunction is present in individuals with prediabetes prior to the development of overt type 2 diabetes.

### CAN and CVD risk

CAN prevalence has been extensively investigated in diabetes and is associated with increased morbidity and mortality risk [48–52]. Therefore, the high morbidity and mortality rates that occurs as a result of CAN is of major concern, given the global epidemic of prediabetes. CAN itself is linked to increased prevalence of silent presentation of myocardial ischaemia [52]. An increase in all-cause mortality rates and cardiovascular events was independently associated with resting and mean heart rate in post hoc analyses of the Ongoing Telmisartan Alone and in combination with Ramipril Global Endpoint Trial (ONTARGET) and the Telmisartan Randomised Assessment Study in ACE Intolerant Subjects with Cardiovascular Disease (TRANSCEND) trial [53]. Both were large trials in medically optimised patients with stable CVD. Importantly, for an increase of 10 beats/min in resting and mean heart rate, there was a significant increase in risk of major vascular events, cardiovascular death, congestive heart failure and all-cause mortality rates [53]. Resting heart rate and blunted HRV are two measures of cardiac autonomic nervous system imbalance, the influence of which were evaluated in the Framingham Heart Study offspring cohort [54]. These measures, in addition to demographic (age and sex) and cardiovascular risk factors (smoking) were significant predictors in the development of CVD, diabetes and premature death (within 12 years) [54]. Given that myocardial blood flow is regulated by cardiac adrenergic signalling and coronary blood flow increases in response to sympathetic stimulation [55], it is of little surprise that CAN results in impaired coronary blood flow [56, 57]. Despite the significant excess CVD events and mortality risk, CAN screening is not routinely performed as a part of annual diabetes or prediabetes screening.

### Pathophysiology of CAN in prediabetes

The pathophysiology of CAN relates to multifactorial changes that occur in prediabetes and lead to oxidative stress, mitochondrial dysfunction with subsequent neuronal damage and dysfunction of autonomic ganglion synaptic transmission [58–60]. Dysglycaemia in prediabetes is a common route to multiple pathophysiological pathways that lead to autonomic neuropathy. Similarly, the findings of Rasic-Milutinovic et al [46] support the notion that continuously elevated glucose level, as a component of the metabolic syndrome, correlates with spectral HRV indices [46]; other supporting data have been published [29]. Interestingly, Rasic-Milutinovic et al [46] also reported disturbed sympathovagal balance with dominant parasympathetic dysfunction in individuals with the metabolic syndrome and type 2 diabetes in keeping with the natural history of CAN. There is an association between BMI, plasma triacylglycerols/remnant lipoproteins and the risk for diabetic peripheral neuropathy even in type 1 diabetes [61]. In a post hoc analysis of participants (*n* = 427) with mild to moderate diabetic neuropathy, elevated triacylglycerols correlated with myelinated fibre density loss independent of disease duration, age, diabetes control or other variables [62]. It has also been suggested that cholesterol-lowering treatments (statins and ezetimibe) [63] and triacylglycerol-lowering treatments (fibrates) [63] may reduce the progression and severity of diabetic peripheral neuropathy. Well-planned randomised trials to evaluate the impact of intensive plasma lipid normalisation on diabetic peripheral neuropathyand CAN are required. Please see Williams et al [23] for a thorough discussion on the multifactorial pathophysiology of CAN in obesity, prediabetes and the metabolic syndrome (including a detailed figure on the pathophysiology). Looking beyond the modest degree of hyperglycaemia, additionally obesity, dyslipidaemia, inflammation and hypertension play a pivotal role in CAN development.

### Prediabetes, IFG or IGT in CAN development

Prediabetes is defined using IFG, IGT or HbA_1c_ and three of the studies in this review showed that these components may have a different effect on the development of CAN. Watkins et al [64] established that the association between autonomic function and IFG was primarily mediated through hypertension, obesity and ageing, suggesting the possibility that elevated fasting glucose has a lesser effect on the impairment of autonomic control. Dimova et al [36] highlighted the role of postprandial hyperglycaemia, with 120 min glucose correlating to sympathetic activity [36]. The MONICA/KORA study investigated the prevalence of CAN in isolated IFG (iIFG), isolated IGT (iIGT) and combined IFG and IGT (IFG+IGT), showing differential prevalence within these groups (iIFG 8.1%, iIGT 5.9%, IFG+IGT 11.4%) [25]. This trend is consistent with the suggestion made by Watkins et al [64] that each of these components may independently contribute to CAN, although the prevalence rates in all prediabetes groups were the lowest of the included studies. Ziegler et al [25] suggest that the individuals with IFG+IGT are at the highest risk of autonomic dysfunction compared with iIGF and iIGT. In a meta-analysis, annualised incidence rates of diabetes for individuals with iIGT (4–6%) and iIFG (6–9%) were lower than those in individuals with IFG+IGT (15–19%) [65]. This may suggest that iIFG and iIGT, albeit they are insulin-resistant states, differ in their pathophysiology and site of insulin resistance. People with iIFG predominantly have hepatic insulin resistance, whereas individuals with iIGT have moderate to severe muscle insulin resistance [66]. Insulin resistance results in oxidative stress through mitochondrial dysfunction, which is characterised by smaller mitochondrial size and decreased mitochondrial DNA content [67]. Indeed, glucose-mediated oxidative stress contributes to the development and progression of diabetic neuropathy by inducing an imbalance in mitochondrial biogenesis and fission [68]. There is also a higher prevalence of obstructive sleep apnoea in prediabetes and the metabolic syndrome [69]. Several studies in individuals with obstructive sleep apnoea have shown autonomic nervous system alterations, in particular sympathetic overactivity, both acutely during apnoea events and chronically during the daytime [70].

### Early detection of CAN

The early detection of CAN is crucial in its treatment due to it being readily reversible in people with prediabetes [71]. Several studies have reported that good glycaemic control reduces the incidence of CAN and slows its progression, particularly in the early stages but not when advanced autonomic abnormalities appear [11, 58, 72]. Additionally, there is a positive correlation between the duration of diabetes and CAN [14]. Consequently, it is important to underline the high prevalence of CAN during the early stages of glycaemic dysregulation where CAN, prediabetes and the metabolic syndrome are all reversible. Treating the modifiable risk factors for prediabetes and the metabolic syndrome that also modulate autonomic dysfunction presents an opportunity for the reversal of CAN. There remains a paucity of data in this cohort (prediabetes/the metabolic syndrome); however, lifestyle intervention in people with IFG or IGT resulted in a reduction in heart rate and increase in HRV over 4 years, according to the Diabetes Prevention Programme [71]. Therefore, lifestyle intervention is an effective means of managing early CAN and should be utilised as a part of a multifactorial therapeutic strategy.

### Limitations and future work

There were a number of causes of high heterogeneity, including participant populations and ethnicity, therefore limiting the study to a systematic review. Ethnicity is a predictor for the development of CAN, thus comparing prevalence rates of CAN between different ethnicities is problematic [45]; however, a recent study found no differences between South Asians and white Europeans in the prevalence of CAN [73]. The final number of articles included in this review was small, which prevented detailed secondary analyses. None of the studies included displayed an appropriate sample size calculation and some had low numbers of participants. Several studies were hospital based and also recruited participants from a single centre while others did not detail recruitment strategies. This study was limited to English language and published data which may introduce bias. Additionally, there was heterogeneity in relation to the method of diagnosis of prediabetes (and new-onset diabetes/NGT), as both HbA_1c_ and glucose tolerance were included as methods for diagnosis. Unfortunately, a detailed analysis of the metabolic syndrome was not possible due to limited numbers of studies. Directions for future research include establishing whether subclinical CAN progresses to overt CAN and examining the relationship of CAN to other microvascular complications in a dedicated prevalence study in the general population incorporating a prospective longitudinal cohort. In addition, with the recent success of the Diabetes Remission Clinical Trial (DiRECT) in achieving diabetes remission through a very-low-energy diet [74], a further interrogation of this effect on the natural history of CAN in a prediabetes population is warranted.

### Conclusion

There is a higher than expected prevalence of CAN in prediabetes. Early detection of CAN through population-level screening needs careful consideration in view of the excess morbidity and mortality risk associated with this condition. However, there is still a need for an adequately sized international multicentre population-based study to ascertain the prevalence of definite CAN with longitudinal follow-up for ‘hard’ cardiovascular outcomes in prediabetes.

## Supplementary Information

ESM (PDF 1.12 MB)

## Data Availability

The datasets generated during and/or analysed during the current study are available from the corresponding author on reasonable request. All data are available from primary publication sources.

## Abbreviations

AFT: Autonomic function test
CAN: Cardiac autonomic neuropathy
HRV: Heart rate variability
IFG: Impaired fasting glucose
IGT: Impaired glucose tolerance
iIFG: Isolated IFG
iIGT: Isolated IGT
NGT: Normal glucose tolerance
