# Supplementary material for: The prevalence of cardiac autonomic neuropathy in prediabetes: a systematic review
Source: Diabetologia. 2020 Nov 9;64(2):288–303. doi: 10.1007/s00125-020-05316-z (PMC7801295; doi:10.1007/s00125-020-05316-z)
Supplement: Supplementary file 1 — (PDF 1.12 MB) [file 125_2020_5316_MOESM1_ESM.pdf]

Extended Supplementary Material

ESM Table 1. CAN study characteristics and prevalence in the metabolic syndrome in final selected articles

**Author**	**Country**	**Sample Size**	**Study group**	**Group Size**	**Mean Age (years +/- SD or range)**	**Sex (Female/ Male)**	**Number of AFTs used for diagnosis (1 or ≥2)**	**AFT tests**	**Number of Subjects with CAN**	**Prevalence (%)**
Kseneva et al [47]	Russia	135	Metabolic Syndrome	135	49.7±0.8	105/30	1	HRV during orthostatic test	33	24.4
Rasic-Milutinovic et al [46]	Serbia	49	Metabolic Syndrome	15	57.63±7	9/6	≥2	Hand grip test; orthostatic hypotension; HRV during deep breathing including E/I ratio; max/min 30/15; ratio to standing up and Valsalva ratio	6	42.9
			NGT	15	55 a	a			a	a
			(T2)DM	17	55.36±4.64	10/7			8	46.2

ESM Table 2. Metabolic characteristics of CAN studies in the metabolic syndrome in final selected articles

Author	Study group	FPG (mmol/L ±SD OR range)	OGTT (mmol/L ±SD OR range)	HbA1C % (mmol/mol)	BP Systolic (mmHg ±SD OR range)	BP Diastolic (mmHg ±SD OR range)	Triglycerides (mmol/L ±SD OR range)	Cholesterol (mmol/L ±SD OR range)	BMI (kg/m^2 ±SD OR range)
Kseneva et al [47]	Metabolic Syndrome	a	a	a	a	a	a	a	a
Rasic-Milutinovic et al [46]	Metabolic Syndrome	6.02±0.54	a	6.10[b] (43.2)	128.43±16.35	78.47±8.42	3.25±1.35	6.54±0.74	28.59±0.75
	NGT	5.02±0.54	a	4.90 [b] (30.1)[b]	125.43±18.46	76.57±5.76	1.15±0.90	5.94±0.74	26.59±1.64
	(T2)DM	6.69±1.14	a	7.20 [b] (55.2)	129.23±15.52	79.08±12.13	2.13±0.59	5.40±0.79	27.77±2.9

Abbreviations: NGT (normal glucose tolerance); DM (Diabetes mellitus); (T2)DM (type 2 Diabetes mellitus)

a: Information are NA, b: Only mean values were available.

Abbreviations: FPG (fasting plasma glucose); NGT (normal glucose tolerance); DM (Diabetes mellitus); (T2)DM (type 2 Diabetes mellitus)

Rasic- Milutinovic et al [46] – HbA1c dispersion data (SD or range) are unavailable.

ESM Table 3. Definition of abnormal AFT

Author	AFT tests	Definition of abnormal AFT
Ziegler et al [25]	Linear HRV analysis (time and frequency domain) and nonlinear HRV analysis derived from nonlinear dynamics	Abnormal indices: <5th or >95th percentile
Dimova et al[41]	ANX- 3.0 and Ewing tests®: deep breathing challenge, Valsalva challenge, and stand-up challenge.	ANX- 3.0 method that computes sympathetic and parasympathetic activity non-invasively, separately and simultaneously based on cardio-respiratory synchronization at rest. There is no universal reference value as ANSAR system uses individual age-based low “cut-off” values above which sympathetic and parasympathetic response during a particular test is normal for the particular examined patient.
Laitinen et al [38]	Deep-breathing test; Active orthostatic test; Expiration ⁄ Inspiration (E⁄ I) ratio	Parasympathetic dysfunction was classified as an E/I ratio of ≤1.10. Sympathetic dysfunction was assessed as decrease of systolic blood pressure ≥ 20 mmHg during standing)
Wu et al [42]	Orthostatic hypotension<br>Blood pressure and heart rate variability after standing	Orthostatic hypotension was defined as a decline in systolic blood pressure of at least 20 mmHg and/or a decline in diastolic blood pressure of at least 10 mmHg after either 1 or 3 min of standing after an individual changed from a supine to a standing position<br>HRV after standing not defined
Zimmerman et al [40]	Expiration/inspiration ratio (E/I)	The 2004 NGT group was chosen as reference, with a mean E/I ratio of 1.27 (95% CI 1.21–1.32). An E/I ratio of <1.5 SD of the mean for the NGT group was considered abnormal

Callaghan et al[44]	Expiration ⁄ Inspiration (E⁄ I)	Abnormal E-to-I ratio: <5th percentile cut-off of lean control subjects
Dimova et al [36]	HRV at rest and during deep breathing, Valsalva challenge, standing challenge	Not defined but states 'at least two abnormal autonomic tests.'
Balbinot et al [43]	HRV tests comprised three spectral indices (in the frequency domain) and four Ewing tests; including the Valsalva manoeuvre, orthostatic test, deep breathing test, and orthostatic hypotension test.	Heart rate variability tests were performed. These tests comprised three spectral indices (in the frequency domain) and four Ewing tests (35), including the Valsalva manoeuvre, orthostatic test, deep breathing test, and orthostatic hypotension test. The electrocardiogram was recorded (particularly the QRS complex) using electrocardiography equipment (Neurosoft®, Ivanovo, Russia) and software that was created for heart rate variability analysis (Poly-Spectrum®).
Dinh et al [37]	HRV at rest; spectral power in the very low-frequency band; spectral power in the low-frequency band; HRV during deep breathing; maximum⁄ minimum 30:15 ratio; Valsalva ratio; postural change in systolic blood pressure	Normal ranges defined previously by Agelink et al based on age and gender [76]
Putz et al [39]	HRV during deep breathing, standing (30/15 ratio) and Valsalva manoeuvre (Valsalva ratio), as well as blood pressure response to standing and sustained handgrip.	Heart rate variability was characterized by the triangular index that is a time domain measure of heart rate variability. It is calculated by a conversion of the RR intervals into a geometric pattern and by dividing the integral of the density distribution with the maximum of the density distribution.
Kamel et al [45]	HRV response to Valsalva manoeuvre Heart-rate variation during deep breathing. Blood pressure response	Using previously defined reference values, the results of the AFT were categorized into one of four groups: normal; possible parasympathetic damage, with results of one of the three tests of parasympathetic function

to standing.<br>Blood pressure response to sustained handgrip was performed by first determining the maximum voluntary contraction using a handgrip dynamometer.	abnormal; definite parasympathetic damage, with results of at least two of the tests of parasympathetic function abnormal; and combined parasympathetic and sympathetic damage, where changes were seen in both arms of testing [77].

ESM Table 4. Critical Appraisal table for assessment of final selected articles

	Callaghan et al 2020 [44]	Dimova 2017 [36]	Dimova et al 2020 [41]	Dihn 2011 [37]	Putz 2013 [39]	Wu 2009 [42]	Balbinot 2012 [43]	Kamel 2014 [45]	Laitinen 2011 [38]	Zimmerman 2018 [40]	Ziegler 2015 [25]
Was the sample representative of the target population?	0	0	0	1	0	0	1	0	0	0	0
Were study participants recruited in an appropriate way?	0	1	0	1	1	0	1	1	1	0	0
Was the sample size adequate?	1	0	1	1	1	0	1	1	0	1	0
Were the study subjects and settings described in detail?	0	0	0	0	0	0	0	0	0	0	0
Was the data analysis conducted with sufficient coverage of the identified sample?	0	0	0	0	0	0	0	0	0	0	0
Were valid methods used for the identification of the condition?	1	0	0	0	0	0	0	0	0	0	0
Was the condition measured in a standard, reliable way for all participants?	0	0	0	0	0	1	0	0	0	0	0

Was there appropriate statistical analysis?	0	0	0	0	0	0	0	0	0	0	0
Was the response rate adequate, and if not, was the low response rate managed appropriately?	0	0	0	0	0	1	0	0	0	0	0
Total	2	1	1	3	2	2	3	2	1	1	0
Risk of bias	low	low	low	low	low	low	low	low	low	low	low

ESM Figure. 1. Funnel plot displaying all final selected studies used to establish possible bias

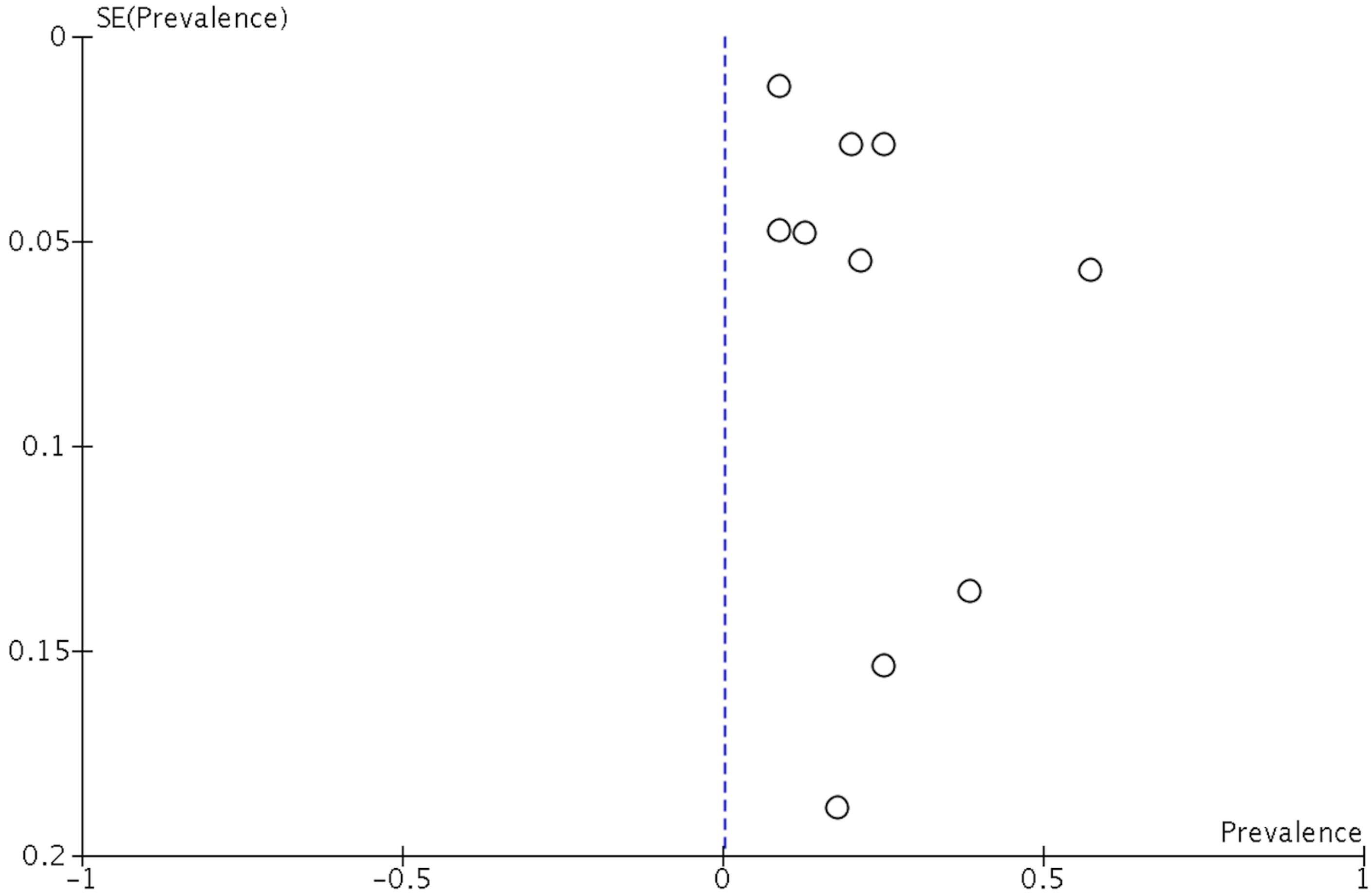

ESM Figure. 2. Forest plots of CAN Prevalence (Expressed as a Proportion) in prediabetes studies a) ≥2 AFT positive studies B) 1AFT positive studies.

A. ≥2 AFT positive studies

Study or Subgroup	CAN Prevalence ≥ 2 tests	SE	CAN Prevalence ≥ 2 tests IV, Random, 95% CI
Balbinot 2012 (H) [43]	0.385	0.135	0.39 [0.12, 0.65]
Dihn 2010 (H) [37]	0.125	0.0477	0.13 [0.03, 0.22]
Dimova 2016 (H+P) [36]	0.198	0.0265	0.20 [0.15, 0.25]
Dimova 2020 (P) [41]	0.086	0.0473	0.09 [-0.01, 0.18]
Kamel 2014 [45]	0.25	0.153	0.25 [-0.05, 0.55]
Ziegler 2015 (P) [25]	0.0859	0.0119	0.09 [0.06, 0.11]

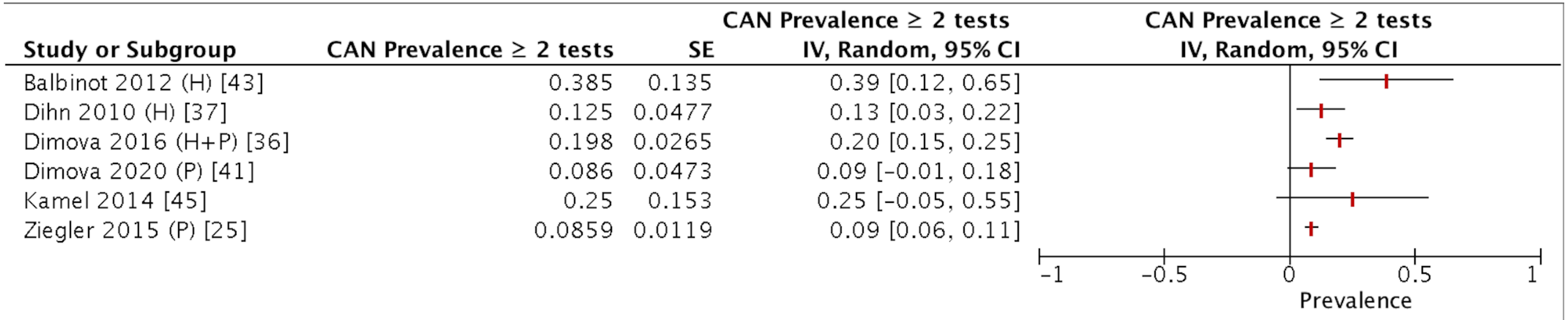

B. 1 AFT positive studies.

Study or Subgroup	CAN prevalence 1 test	SE	CAN prevalence 1 test IV, Random, 95% CI
Callaghan 2020 (H) [44]	0.214	0.0548	0.21 [0.11, 0.32]
Laitinen 2011 (P) [38]	0.25	0.0265	0.25 [0.20, 0.30]
Putz 2012 (H) [39]	0.573	0.0571	0.57 [0.46, 0.68]
Wu 2009 (P) [42]	0.1777	0.0188	0.18 [0.14, 0.21]
Zimmerman 2018 (P) [40]	0	0	Not estimable

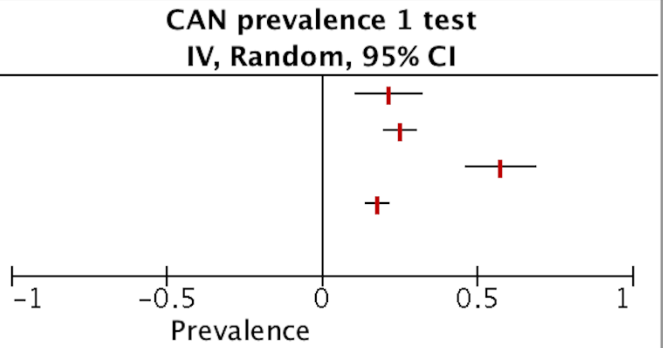

H = Hospital-based, P = Population/Primary Care-based, H+P = Both hospital and primary care based.

*For two studies (Bablinot 2012 and Kamel 2014) with sample sizes of ≤13 and ≤5 people with CAN, the 95% confidence intervals around the prevalence estimate were wide and the lower bound of the confidence interval was estimated to be less than 0. This is due to the small numbers used in the estimation

of prevalence and the standard error (SE) of prevalence (where SE = square root [p(1-p)/n], where p is the prevalence of CAN as a proportion and n is the total number of people in the study or study group), and the uncertainty of the SE is reflected within the wide confidence intervals. The lower bounds of these negative confidence intervals should be considered to be 0.

ESM Figure. 3. Forest plot showing CAN prevalence in NGT studies

Study or Subgroup	CAN Prevalence in NGT	SE	CAN Prevalence in NGT IV, Random, 95% CI
Balbinot 2012 (H) [43]	0.081	0.0449	0.08 [-0.01, 0.17]
Callaghan 2020 (H) (Lean NGT) [44]	0.044	0.0302	0.04 [-0.02, 0.10]
Callaghan 2020 (H) (Obese NGT) [44]	0.182	0.0672	0.18 [0.05, 0.31]
Dimova 2016 (H+P) [36]	0.123	0.0288	0.12 [0.07, 0.18]
Dimova 2020 (P) [41]	0.057	0.0392	0.06 [-0.02, 0.13]
Putz 2012 (H) [39]	0	0	Not estimable
Wu 2009 (P) [42]	0.138	0.0106	0.14 [0.12, 0.16]
Ziegler 2015 (P) [25]	0.045	0.00872	0.04 [0.03, 0.06]
Zimmerman 2018 (P) [40]	0	0	Not estimable

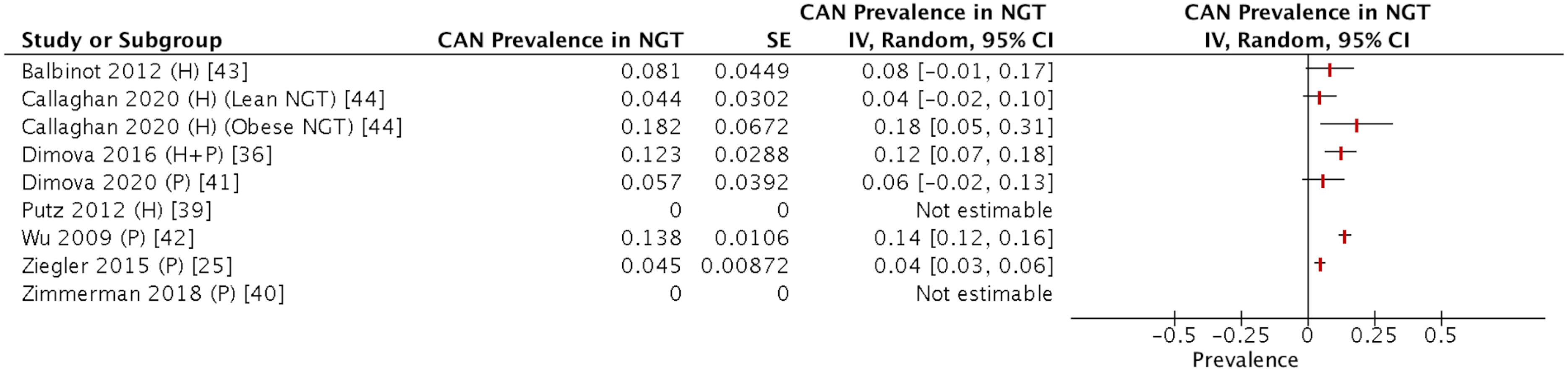

H = Hospital-based, P = Population/Primary Care-based, H+P = Both hospital and primary care based.

*For three studies Bablinot 2012, Callaghan 2020 and Dimova 2020, the 95% confidence intervals around the prevalence estimate were wide and the lower bound of the confidence interval was estimated to be less than 0. This is due to the small numbers used in the estimation of prevalence and the standard error (SE) of prevalence (where SE = square root [p(1-p)/n], where p is the prevalence of CAN as a proportion and n is the total number of people in the study or study group), and the uncertainty of the SE is reflected within the wide confidence intervals. The lower bounds of these negative confidence intervals should be considered to be 0.

ESM Figure. 4. Forest plot showing CAN prevalence in DM studies

Study or Subgroup	CAN prevalence in DM	SE	CAN prevalence in DM IV, Random, 95% CI
Balbinot 2012 (H) [43]	0.552	0.0923	0.55 [0.37, 0.73]
Callaghan 2020 (H) [44]	0.319	0.0665	0.32 [0.19, 0.45]
Dihn 2010 (H) [37]	0.29	0.0629	0.29 [0.17, 0.41]
Dimova 2016 (H+P) [36]	0.322	0.0425	0.32 [0.24, 0.41]
Dimova 2020 (P) [41]	0.235	0.102	0.23 [0.04, 0.43]
Kamel 2014 [45]	0.278	0.106	0.28 [0.07, 0.49]
Wu 2009 (P) [42]	0.255	0.0348	0.26 [0.19, 0.32]
Ziegler 2015 (P) [25]	0.154	0.025	0.15 [0.11, 0.20]
Zimmerman 2018 (P) [40]	0	0	Not estimable

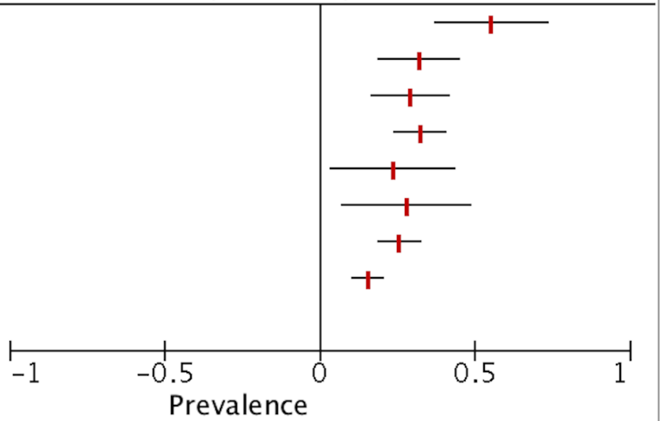

H = Hospital-based, P = Population/Primary Care-based, H+P = Both hospital and primary care based.
